# Novel Rickettsia in Ticks, Tasmania, Australia

**DOI:** 10.3201/eid1510.090799

**Published:** 2009-10

**Authors:** Leonard Izzard, Stephen Graves, Erika Cox, Stan Fenwick, Nathan Unsworth, John Stenos

**Affiliations:** Australian Rickettsial Reference Laboratory, Geelong, Victoria, Australia (L. Izzard, S. Graves, J. Stenos); Murdoch University, Murdoch, Western Australia, Australia (L. Izzard, S. Fenwick, J. Stenos); Launceston General Hospital, Launceston, Tasmania, Australia (E. Cox); Texas A&M Health Science Center, College Station, Texas, USA (N. Unsworth)

**Keywords:** Rickettsia, Candidatus Rickettsia tasmanensis, Australia, Tasmania, Ixodes, ticks, dispatch

## Abstract

A novel rickettsia was detected in *Ixodes tasmani* ticks collected from Tasmanian devils. A total of 55% were positive for the citrate synthase gene by quantitative PCR. According to current criteria for rickettsia speciation, this new rickettsia qualifies as *Candidatus* Rickettsia tasmanensis, named after the location of its detection.

In Australia, 4 rickettsial species are known to cause disease in humans; none of these species has been identified in Tasmania. However, 3 cases of human rickettsial infections in Tasmania have been documented (*1**–**3*). *Ixodes tasmani* ticks are of particular interest because they are known to be vectors for other rickettsial species in Australia (*4*) and are also the most common tick species in Tasmania (*5*). In addition, because these ticks bite humans, they are candidates for rickettsial transmission in Tasmania.

Although *Candidatus* Rickettsia tasmanensis, a proposed new species of rickettsiae, has not been associated with human disease, the possible virulence of this rickettsia cannot be disregarded. Some initially identified rickettsiae were later found to cause disease in humans. For example, *R*. *parkeri* was discovered in 1939 (*6*) but was only confirmed as a human pathogen in 2004 (*7*). To investigate infections in Tasmania, we collected ticks from Tasmanian devils (*Sacrophilus* *harrissi*) and analyzed them for rickettsial species.

## The Study

Forty-four *I*. *tasmani* ticks were collected from Tasmanian devils from various sites in Tasmania during 2005–2006; 36 were engorged females, 5 were nymphs, and 3 were males. Each tick was washed in 70% ethanol, rinsed in sterile phosphate-buffered saline, and homogenized. Homogenates were then subjected to DNA extraction by using a QIAmp DNA Blood Mini Kit (QIAGEN, Hilden, Germany). The presence of a rickettsial agent was detected by real-time PCR (*8*). Characterization of novel rickettsial species was achieved by comparing sequences of genes as described (*9*).

Amplification and sequencing of 1,096-, 3,005-, 588-, and 4,918-bp products for the citrate synthase (*gltA*), surface cell antigen (*sca4*), outer membrane protein A (*ompA*), and *ompB* genes, respectively, were conducted by using primers previously described (*9*). The 16S rRNA (*rrs*) gene was not amplified because cell culture isolation was not performed. Amplicons were cloned by using the TA Cloning Kit (Invitrogen, Carlsbad, CA, USA) and extracted by using a QuickLyse Mini Prep Kit (QIAGEN).

Big Dye sequencing was performed by using a GeneAmp PCR System 2400 thermocycler (Applied Biosystems, Foster City, CA, USA). Resulting products were analyzed at the Australian Genomic Research Facility by using an ABI Prism 3730xl DNA Analyzer (Applied Biosystems).

Sequences were assembled and edited by using the SeqMan Pro program (DNASTAR, Inc., Madison, WI, USA) and evaluated by using neighbor-joining and maximum-parsimony methods in MEGA 4 (*10*) and the maximum-likelihood method in PHYLIP (*11*). Results were confirmed by using BLAST analysis software (http://blast.ncbi.nlm.nih.gov/Blast.cgi). All sequences have been deposited in GenBank (Table).

**Table:** GenBank accession numbers of additional rickettsia sequences used in this study*

Strain	*rrs*	*gltA*	*ompA*	*ompB*	*sca4*
*Rickettsia* sp. 518	–	EU430246	EU430247	EU430242	–
*Candidatus* R. tasmanensis T120	–	GQ223395	–	GQ223396	GQ223397
*Candidatus* R. tasmanensis T152-E	–	GQ223391	GQ223392	GQ223393	GQ223394

*rrs, 16S rRNA; *gltA*, citrate synthase; *omp*, outer membrane protein; *sca*, surface cell antigen.

**Figure 1:**
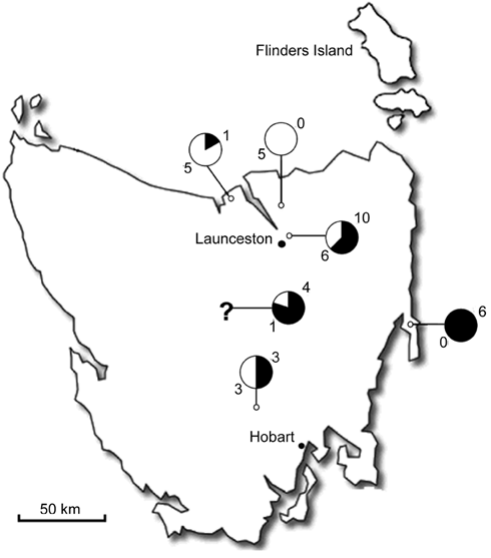
Map of Tasmania, Australia, showing number of positive (black) and negative (white) ticks and their locations. The question mark indicates unknown locations. A total of 55% of the ticks were positive for a spotted fever group rickettsia.

Rickettsial DNA was detected in 24 (55%) of 44 *I*. *tasmani* ticks by using a *gltA*-specific quantitative PCR (qPCR) assay. Because most ticks were engorged females, no statistical correlation was found between the sex of ticks and presence of rickettsiae. Distribution of the ticks collected and degree of positivity are shown in Figure 1.

Sequences from *I*. *tasmani* ticks were compared with reported sequences (*12*). Results showed that the closest phylogenetic relative for 3 of the genes was *R*. *raoultii* strain Khabarovsk, with sequence similarities of 99.1% (1,086 bp/1,096 bp), 96.9% (570 bp/588 bp), and 97.7% (4,782 bp/4,895 bp) for the *gltA*, *ompA*, and *ompB* genes, respectively, and 98.1% (2,930 bp/2,988 bp) to *R*. *japonica* strain YM for the *sca4* gene.

Comparison of our sequences with that of a partially sequenced rickettsia (*R*. *tasmanensis* strain T120) previously detected in an *I*. *tasmani* tick removed from a child near Underwood, Tasmania (N. Unsworth, unpub. data) found homology levels to be within the species threshold. No data on the clinical state of the child were obtained.

Sequences closely matched genes of a second partially sequenced rickettsia (*Rickettsia* sp. 518) from an *I*. *tasmani* tick removed from a Tasmanian devil in Tasmania by researchers at Macquarie University (Sydney, New South Wales, Australia) (*13*). Of the 3 partial gene sequences reported, *ompB* and *gltA* gene sequences matched to the species level with *Candidatus* R. tasmanensis; however, *ompA* gene sequences did not. Their isolate could be another new species, although it is difficult to draw conclusions with sequences of small fragments.

**Figure 2:**
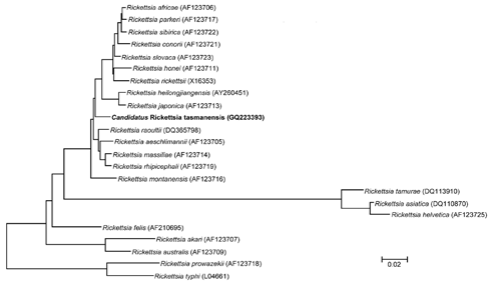
Phylogenetic tree showing the relationship of a 4,834-bp fragment of the outer membrane protein B gene of *Candidatus* Rickettsia tasmanensis (in **boldface**) among all validated rickettsia species. The tree was prepared by using the neighbor-joining algorithm within the MEGA 4 software (*10*). Bootstrap values are indicated at each node. Scale bar indicates 2% nucleotide divergence.

Results of sequence analysis of the *ompB* gene by using the neighbor-joining algorithm are shown in Figure 2. Although all selected genes were analyzed, the *ompB* gene tree had the strongest bootstrap values and the largest compared fragment size.

## Conclusions

All 44 ticks were collected from northeastern and eastern Tasmania. The number of positive samples (55%) contrasts with the small number of reported SFG rickettsial human infections in Tasmania because *I*. *tasmani*, which is known to opportunistically bite humans (*5*), has a high density in Tasmania. Clinical cases of infection may be missed because physicians are not aware of human rickettsial disease in Tasmania.

A recent study showed a high level of exposure to SFG rickettsia in cats and dogs near the city of Launceston, Tasmania (L. Izzard, unpub. data); in the Ravenswood area 10 of 16 tick samples were positive for SFG rickettsia by qPCR. However, the species of SFG rickettsiae could not be determined in this study because only serologic analysis was conducted. Because *I*. *tasmani* ticks are common in Tasmania and parasitize cats and dogs, *Candidatus* R. tasmanensis is likely to be the causative agent in some of the cases.

When gene sequences of *Candidatus* R. tasmanensis were compared with those of validated species (*12*), they did not closely match either of the 2 validated SFG rickettsia in Australia (*R*. *australis* or *R*. *honei*). Similarly, *Candidatus* R. tasmanensis sequences were divergent from 2 *Candidatus* species (*Candidatus* R. gravesii and *Candidatus* R. antechini) in Australia, which are currently being characterized. *Candidatus* R. tasmanensis had the highest phylogenetic similarity to *R*. *raoultii* strain Khabarovsk for 3 of 4 gene sequences. This rickettsial species was isolated in the Russian Far East (>10,000 km north of Tasmania) from a *Dermacentor silvarum* tick and is a known human pathogen (*12*). However, the similarities between the gene sequences of these 2 organisms were well below the threshold defined by Fournier et al. (*9*). On the basis of these results, we propose to give this *Rickettsia* sp. a *Candidatus* status and formally name it *Candidatus* R. tasmanensis after the location from which it was originally isolated. To validate *Candidatus* R. tasmanensis as a novel species, isolation and subsequent sequencing of its *rrs* gene are needed. Multigene sequencing of 4 other qPCR-positive *I*. *tasmani* ticks will also be useful.

Because the range of this study was limited to eastern Tasmania, *I*. *tasmani* ticks from western Tasmania and other parts of Australia should also be examined for this rickettsial agent. This analysis would help determine its true range. Testing the blood of animals infested with *I*. *tasmani* ticks for evidence of SFG rickettsial exposure may also provide data on the pathogenesis and range of this rickettsia.

## References

[1] Unsworth NB, Stenos J, Graves SR, Faa AG, Cox GE, Dyer JR, Flinders Island spotted fever rickettsioses caused by “marmionii” strain *Rickettsia honei*, eastern Australia. Emerg Infect Dis. 2007;13:566–73. 10.3201/eid1304.060087 17553271 PMC2725950

[2] Chin RH, Jennens ID. Rickettsial spotted fever in Tasmania. Med J Aust. 1995;162:669. 7603385 10.5694/j.1326-5377.1995.tb126063.x

[3] Watts MR, Benn RA, Hudson BJ, Graves S. A case of prolonged fatigue following an acute rickettsial infection. QJM. 2008;101:591–3. 10.1093/qjmed/hcn064 18474521

[4] Sexton DJ, Dwyer B, Kemp R, Graves S. Spotted fever group rickettsial infections in Australia. Rev Infect Dis. 1991;13:876–86. 1962102 10.1093/clinids/13.5.876

[5] Roberts FH. Australian ticks. Melbourne: Commonwealth Scientific and Industrial Research Organisation; 1970.

[6] Parker R, Kohls G, Cox G, Davis G. Observations on an infectious agent from *Amblyomma maculatum.* Public Health Rep. 1939;54:1482–4.

[7] Paddock CD, Sumner JW, Comer JA, Zaki SR, Goldsmith CS, Goddard J, *Rickettsia parkeri*: a newly recognized cause of spotted fever rickettsiosis in the United States. Clin Infect Dis. 2004;38:805–11. 10.1086/381894 14999622

[8] Stenos J, Graves S, Unsworth N. A highly sensitive and specific real-time PCR assay for the detection of spotted fever and typhus group rickettsiae. Am J Trop Med Hyg. 2005;73:1083–5. 16354816

[9] Fournier PE, Dumler JS, Greub G, Zhang J, Wu Y, Raoult D. Gene sequence-based criteria for identification of new *Rickettsia* isolates and description of *Rickettsia heilonjiangensis* sp. nov. J Clin Microbiol. 2003;41:5456–65. 10.1128/JCM.41.12.5456-5465.2003 14662925 PMC308961

[10] Tamura K, Dudley J, Nei M, Kumar S. MEGA4: Molecular Evolutionary Genetics Analysis (MEGA) software version 4.0. Mol Biol Evol. 2007;24:1596–9. 10.1093/molbev/msm092 17488738

[11] Felsenstein J. PHYLIP: Phylogeny Inference Package (Version 3.2). Cladistics. 1989;5:164–6.

[12] Mediannikov O, Matsumoto K, Samoylenko I, Drancourt M, Roux V, Rydkina E, *Rickettsia raoultii* sp. nov., a spotted fever group rickettsia associated with *Dermacentor* ticks in Europe and Russia. Int J Syst Evol Microbiol. 2008;58:1635–9. 10.1099/ijs.0.64952-0 18599708

[13] Vilcins IM, Old JM, Deane E. Detection of a *Hepatozoon* and spotted fever group *Rickettsia* species in the common marsupial tick (*Ixodes tasmani*) collected from wild Tasmanian devils (*Sarcophilus harrisii*), Tasmania. Vet Parasitol. 2009;162:23–31. 10.1016/j.vetpar.2009.02.015 19303711

